# Organic room temperature phosphorescence co‐crystal with reversible acid/base stimulus response

**DOI:** 10.1002/smo.20240054

**Published:** 2025-01-04

**Authors:** Chenchen Zhang, Xingjia Jiang, Can Wang, Zhaoyang Liu, Bin Xu, Wenjing Tian

**Affiliations:** ^1^ State Key Laboratory of Supramolecular Structure and Materials Jilin University Changchun Jilin Province China

**Keywords:** co‐crystal, reversible, room temperature phosphorescence (RTP), stimulus response

## Abstract

Stimulus‐responsive organic room temperature phosphorescent (RTP) materials have received significant attention in bioimaging, sensing, and data storage because of their controllable dynamic variability and rapid response. Organic co‐crystals, with tailor‐designed optical properties through manipulation of their aggregate structures, have proven to be very effective in elucidating the structure‐property relationship of organic RTP materials at the molecular level. Therefore, enhancing RTP through rigid frameworks that promote intersystem crossing is a valid approach. Notably, the realization of organic RTP co‐crystal performance by altering the components or adjusting the crystal lattices is highly appealing; however, this has not been fully addressed. In this study, an organic RTP co‐crystal, 4,4′‐bipyridine (44BD), was employed as the host, and 1,4‐diiodotetrafluorobenzene (DITF) and 4‐bromo‐2,3,5,6‐tetrafluorobenzoic acid (TFBA) were employed as guests. The 44BD‐DITF co‐crystal exhibited an orange RTP, whereas 44BD‐TFBA displayed a bright yellow RTP. Crystal analysis and theoretical calculations revealed that dense molecular packing and abundant intermolecular interactions within these co‐crystals are crucial for the emergence of RTP. Notably, both co‐crystals show a reversible acid/base stimulus response, that is, exposure to hydrochloric acid (HCl) fumes results in quenching of their RTP, which can be subsequently restored by triethylamine (TEA) fumigation. This study presents an effective approach towards reversible RTP switching in organic co‐crystals, thus offering opportunities for the development of acid/base stimulus‐responsive materials for next‐generation applications.

## Graphical abstract

This work designed and prepared two stimulus—responsive room temperature phosphorescent co‐crystals named 4,4′‐bipyridine‐1,4‐diiodotetrafluorobenzene (44BD‐DITF) and 44BD‐tetrafluorobenzoic acid (TFBA) with 44BD as the main body and two organic aromatic small molecules DITF and TFBA as the guest. Furthermore, both co‐crystals exhibit obvious RTP properties and efficient acid/base stimulus response properties by alternately applying the HCl and TEA fumigation.

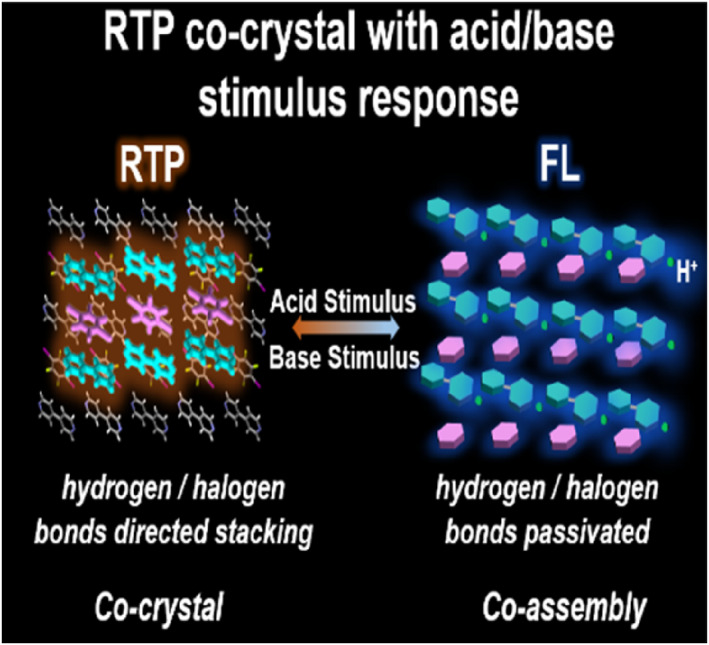

## 1 INTRODUCTION

Organic room‐temperature phosphorescent (RTP) materials have attracted significant interest owing to their significant advantages, such as large Stokes shifts[^1^, ^2^, ^3^] ultralong lifetimes,^[4]^ and efficient utilization of the excited state energy.^[5]^ By employing strategies involving heteroatoms[^6^, ^7^] heavy atom,[^8^, ^9^]and multimers[^10^, ^11^] a substantial number of organic luminophores exhibiting long‐lived phosphorescence under ambient conditions have been discovered and realized.[^12^, ^13^]

Recent advances in organic RTP materials have highlighted the critical role of molecular orientation and stacking modes in their luminescent properties.^[14]^ Pioneering research has delicately incorporated guest molecules into host organic crystals via halogen or hydrogen bonds to create co‐crystal.[^15^, ^16^] These organic co‐crystals offer the ability to finely tune physicochemical properties through the design and control of molecular stacking modes and noncovalent intermolecular interactions. Notably, co‐crystal has successfully modulated RTP performance by altering the components or modifying crystal lattice.[^17^, ^18^] Further elucidation of the structure‐property relationship of organic RTP materials at the molecular level is highly appealing, yet has not been fully addressed. In particular, developing stimulus‐responsive RTP materials that are sensitive to variations in environmental information may lead to a new library of intelligent materials because the weak halogen‐^[19]^ and hydrogen bonding[^20^, ^21^]in supramolecular co‐crystals are highly susceptible to external stimuli, such as pH[^22^, ^23^] heat[^24^, ^25^] and mechanical force[^26^, ^27^] resulting in dynamic changes in emission color,^[28]^ intensity,^[29]^ and lifetime.^[25]^

Strongly acidic or base environments are typically detrimental to life^[30]^; hence, highly sensitive detection of acids/bases is a crucial research area. To date, most acid/base‐responsive organic materials are based on fluorophores.^[31]^ Organic RTP co‐crystals, which are sensitive to acidic/base conditions, show significant promise for practical applications owing to their good reversibility,^[32]^ rapid response,^[33]^ and ease of control.^[34]^ Feng and coworkers fabricated the novel “pyrene box” cocrystals, which have a reversible transition between fluorescence and phosphorescence states with the alternating fuming of triethylamine (TEA) and hydrochloric acid (HCl) gases.^[35]^ In addition, Li et al. introduced a pyridine group into the guest molecule of the co‐crystals, leading to multistage HCl/NH_3_‐responsive RTP characteristics.^[36]^ Although the above RTP co‐crystals showed promising responses to acid/base, a molecular‐level understanding of the factors dominating the packing structures during the acid/base stimulus‐RTP process is urgently needed.

Herein, we demonstrate two acid/base stimulus‐responsive RTP co‐crystals with 4.4 ′‐ bipyridine(44BD) as the host and 1,4‐diiodotetrafluorobenzene (DITF)/4‐bromo‐2,3,5,6‐ tetrafluorobenzoic acid (TFBA) as the guest. The pure host molecule showed negative RTP performance. Meanwhile, the two guest molecules could induce multiple halogen bonds in the co‐crystal lattices, which successfully boosted the RTP emission effect by enhancing the ISC process. Remarkably, the intersystem crossing rate of 44BD‐TFBA co‐crystal reaches 3.68 × 10^10^ s^−1^, compared to the initial 2.30 × 10^8^ s^−1^ for pure 44BD crystal. Additionally, both co‐crystals can exhibit reversible acid/base stimulus RTP responses owing to the destruction/formation of intermolecular halogen bonds, which are essential for the further development of smart materials.

## 2 RESULTS AND DISCUSSION

**FIGURE 1:**
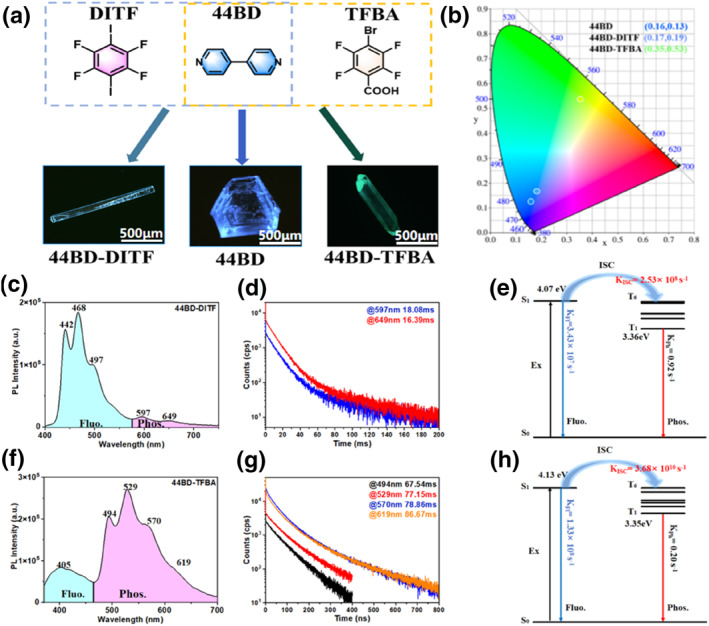
(a) Molecular structures and images of the 44BD crystal and 44BD‐DITF/44BD‐TFBA co‐crystals under UV light. (b) Corresponding positions of (co)crystals in CIE chromaticity coordinates. (c) Photoluminescence spectra of 44BD‐DITF co‐crystal. (d) Phosphorescent lifetime of 44BD‐DITF co‐crystal at 597 and 649 nm. (e) Theoretically calculated energy levels of the 44BD‐DITF co‐crystal. (f) Photoluminescence spectra of the 44BD‐TFBA co‐crystal. (g) Phosphorescent lifetimes of the 44BD‐TFBA co‐crystal at 494, 529, 570, and 619 nm. (h) Theoretically calculated energy levels of the 44BD‐TFBA cocrystal. 44BD, 4,4′‐bipyridine; DITF, 1,4‐diiodotetrafluorobenzene; TFBA, tetrafluorobenzoic acid.

The 44BD crystal and 44BD‐DITF/44BD‐TFBA co‐crystals were obtained using the conventional solvent evaporation method. The adsorption spectra of the co‐crystals have an obvious red shift based on the participation of guest molecules, which indicates a better planarity of the 44BD conformation (Supporting Information S1: Figure S1). Pure 44BD crystals showed typical blue fluorescence with an emission peak at 445 nm under 365 nm ultraviolet excitation (Figure 1b and Supporting Information S1: Figure S1), while co‐crystal formation led to obvious changes in fluorescence properties. The 44BD‐DITF co‐crystals showed enhanced fluorescence emission at 442, 468, and 497 nm (Figure 1c and Supporting Information S1: Figure S2), and the fluorescence efficiency (Φ_Fl_) increased from 9.65% (44BD crystal) to 11.93%. However, the fluorescence of the 44BD‐TFBA co‐crystal blue‐shifted to 405 nm with a greatly reduced Φ_Fl_ of 0.36% (Supporting Information S1: Table S1). Both the co‐crystals had excellent crystallinity, as shown in the XRD results in Supporting Information S1: Figure S3. Moreover, the phosphorescence properties of the three crystals were investigated. Despite the 44BD crystal having no RTP performance, the formation of 44BD‐DITF and 44BD‐TFBA co‐crystals can efficiently turn on the RTP emission. After the removal of UV irradiation, the 44BD‐DITF co‐crystal appeared as an orange afterglow, whereas the 44BD‐TFBA co‐crystal appeared as a yellow afterglow. As illustrated in Figure 1c,d, the 44BD‐DITF co‐crystal has two phosphorescence emission peaks located at 597 and 649 nm, with a lifetime of 8.08 and 16.39 ms, respectively. Interestingly, the phosphorescence intensity of the 44BD‐TFBA co‐crystal was substantially improved, as the emission peaks located at 494, 529, 570, and 619 nm had lifetimes of 67.54, 77.15, 78.86, and 86.67 ms, respectively (Figure 1f,g). The corresponding energy levels were calculated to further explain the different RTP properties of the 44BD‐DITF and 44BD‐TFBA co‐crystals, as shown in Figure 1e,h. The intersystem crossing rate of the 44BD‐TFBA co‐crystal (3.68 × 10^10^ s^−1^) was two orders of magnitude higher than that of the 44BD‐DITF co‐crystal (2.53 × 10^8^ s^−1^), indicating an enhanced ISC process in the 44BD‐TFBA co‐crystal. The intersystem transition efficiency (Φ_ISC_) and nonradiative transition rate (*k*_Ph, nr_) are summarized in Supporting Information S1: Tables S1 and S6. Compared to the 44BD‐DITF co‐crystal (Φ_ISC_ = 88.07%, *k*_Ph, nr_ = 54.39 s^−1^), the 44BD‐TFBA co‐crystal has a higher Φ_ISC_ and a lower *k*_Ph, nr_ (Φ_ISC_ = 99.6%, *k*_Ph, nr_ = 12.76 s^−1^), better explaining the higher intensity of RTP emission in the 44BD‐TFBA co‐crystal. Moreover, the phosphorescence rate (*k*_ph_) is the key factor related to the RTP lifetime, and the smaller *k*_ph_ for the 44BD‐TFBA co‐crystal (0.20 s^−1^) demonstrates a longer afterglow.

**FIGURE 2:**
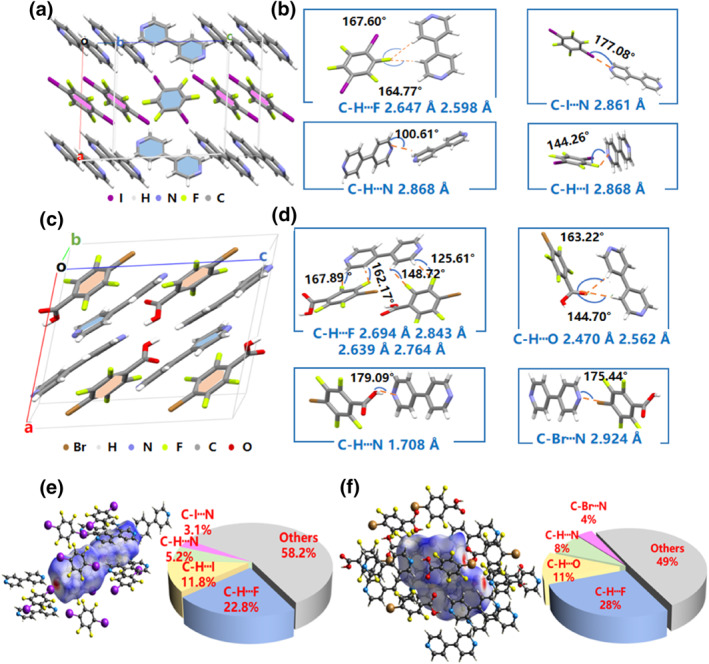
(a) Single‐unit cell structure of the 44BD‐DITF co‐crystal. (b) Typical hydrogen and halogen bond interactions in the 44BD‐DITF co‐crystal. (c) Single‐unit cell structure of 44BD‐TFBA co‐crystal. (d) Typical hydrogen and halogen bond interactions in 44BD‐TFBA co‐crystal. (e) Hirschfeld surface analysis of intermolecular interactions in the 44BD‐DITF co‐crystal. (f) Hirschfeld surface analysis of intermolecular interactions in the 44BD‐TFBA co‐crystal. 44BD, 4,4′‐bipyridine; DITF, 1,4‐diiodotetrafluorobenzene; TFBA, tetrafluorobenzoic acid.

Single‐crystal structures and non‐covalent intermolecular interactions were examined to determine the origin of the RTP phenomenon (Supporting Information S1: Table S2). As shown in Figure 2a and Supporting Information S1: Figure S5, the packing ratio of 44BD and DITF was 1:1 in the co‐crystal, and these two molecules stacked in separate columns along the b‐axis. The majority of intermolecular interactions in the 44BD‐DITF co‐crystal are summarized in Figure 2b, including C–I···N halogen bonds with a distance of 2.861 Å, C–H···I hydrogen bonds with a distance of 2.868 Å, C‐H···N hydrogen bonds with a distance of 2.868 Å, and double C–H···F hydrogen bonds with distances of 2.598 Å/2.647 Å (Supporting Information S1: Table S4). However, the 44BD and TFBA molecules stack in an interdigitated mode along the c‐axis with a packing ratio of 1:1 in the 44BD‐TFBA co‐crystal unit lattice (Figure 2c and Supporting Information S1: Figure S5). Quadruple C‐H···F hydrogen bonds were investigated between two TFBA molecules and one 44BD molecule with distances of 2.694, 2.843, 2.639, and 2.764 Å. Moreover, the double C‐H···O hydrogen bond (2.470 Å/2.562 Å), C‐H···N hydrogen bond (1.708 Å), and C‐Br···N halogen bond (2.924 Å) were also dominant interactions (Figure 2d and Supporting Information S1: Table S5).

Compared with the molecular C‐H···N hydrogen bonds in the pure 44BD crystal, the intermolecular interactions in the 44BD‐DITF and 44BD‐TFBA co‐crystals were much more diversified, which was further analyzed by Hirshfeld surface analysis. The red spots in Figure 2e,f refer to the stronger interaction locations in the co‐crystal structures, which are denoted as C‐I···N halogen bonds in the 44BD‐DITF co‐crystal and C‐Br···N halogen bonds/C‐H ···N hydrogen bonds in the 44BD‐TFBA co‐crystal. Statistical results show that the proportion of hydrogen/halogen bonds in the overall intermolecular interactions is higher in the 44BD‐TFBA co‐crystal. The rich hydrogen/halogen bonds can efficiently suppress the nonradiative transition and enhance the intersystem crossing process. Meanwhile, the phosphorescence radiative rate (*k*_Ph_) could compete with the corresponding non‐radiative rate (*k*_Ph,nr_) at room temperature, and RTP emission would appear. Compared to the 44BD‐DITF co‐crystal (*k*_Ph_ = 0.92 s^−1^, *k*_Ph, nr_ = 54.39 s^−1^), the 44BD‐TFBA co‐crystal had a lower *k*_Ph_ (0.20 s^−1^) and *k*_Ph, nr_ (12.76 s^−1^). Thus, the 44BD‐TFBA co‐crystal displayed a higher phosphorescence intensity and a longer lifetime than the 44BD‐DITF co‐crystal.

**FIGURE 3:**
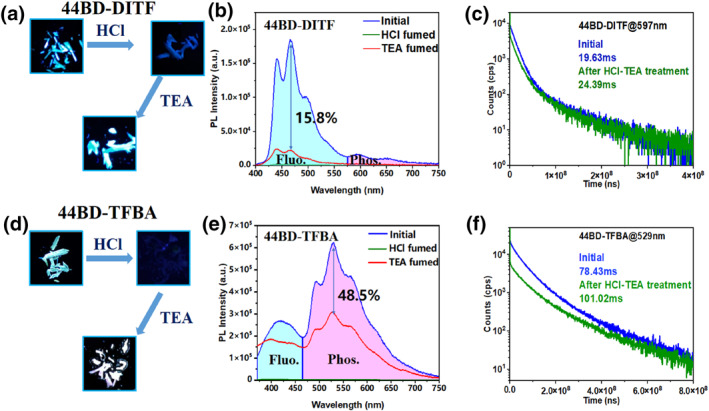
(a) Images of the 44BD‐DITF co‐crystal under illumination with 365 nm UV light before and after hydrochloric acid (HCl)/triethylamine (TEA) treatment. (b) Photoluminescence spectra of 44BD‐DITF co‐crystal before and after HCl/TEA treatment. (c) Phosphorescence lifetime of the 44BD‐DITF co‐crystal at 597 nm before and after HCl/TEA treatment. (d) Images of the 44BD‐TFBA co‐crystal under illumination with 365 nm UV light before and after HCl/TEA treatment. (e) Photoluminescence spectra of 44BD‐TFBA co‐crystal before and after HCl/TEA treatment. (f) Phosphorescence lifetime of the 44BD‐TFBA co‐crystal at 529 nm before and after HCl/TEA treatment. 44BD, 4,4′‐bipyridine; DITF, 1,4‐diiodotetrafluorobenzene; TFBA, tetrafluorobenzoic acid.

Figure 3a,d indicate that both 44BD‐DITF and 44BD‐TFBA co‐crystals exhibit reversible responses under alternating fuming with TEA and HCl gases. After treatment with HCl, RTP was completely quenched in both co‐crystals, and the fluorescence intensity decreased by 99%. Interestingly, the fluorescence and phosphorescence performance of the co‐crystals can be partly recovered with subsequent TEA fumigation. The intensity of the photoluminescence spectra was recovered by 15.8% for the 44BD‐DITF co‐crystal and 48.5% for the 44BD‐TFBA co‐crystal, as portrayed in Figure 3b,e. Moreover, the emission peak positions also shifted back to the initial positions, indicating that the packing motifs were not damaged during HCl/TEA treatment. Compared with initial ones, the HCl/TEA treated co‐crystals have even longer RTP lifetime, which is 24.39 ms for 44BD‐DITF co‐crystal at 597 nm, and 101.02 ms for 44BD‐TFBA co‐crystal at 529 nm.

**FIGURE 4:**
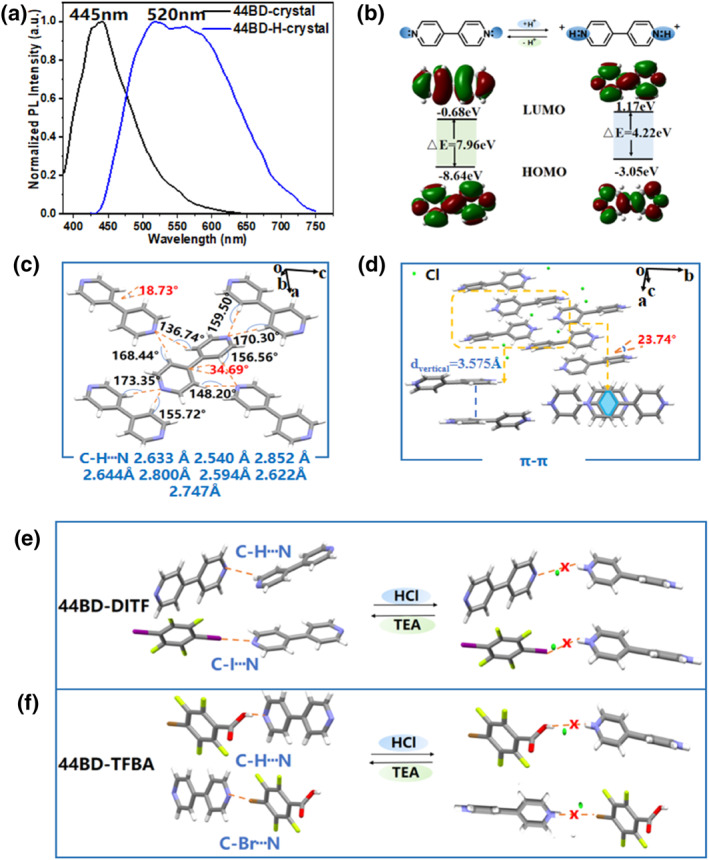
(a) Fluorescence spectra of 44BD and 44BD‐H crystals. (b) Calculated HOMO/LUMO and band gaps of the 44BD and 44BD‐H crystals. (c) Packing patterns and key intermolecular interactions in 44BD crystal. (d) Packing patterns and key intermolecular interactions in 44BD‐H crystal. (e and f) Schematic diagram of the changes in intermolecular interactions with the stimulation of hydrochloric acid (HCl) and triethylamine (TEA) in the 44BD‐DITF co‐crystal (e) and 44BD‐TFBA co‐crystal (f). 44BD, 4,4′‐bipyridine; DITF, 1,4‐diiodotetrafluorobenzene; TFBA, tetrafluorobenzoic acid.

As the pyridine group shows a stimulus response behavior to proton acid,^[25]^ protonated 44BD (44BD‐H) single crystals were synthesized to explore the mechanism of the tunable RTP phenomenon. A comparison of the photoluminescence spectra of the 44BD‐H and 44BD crystals showed that the fluorescence emission peak red‐shifted from 445 to 520 nm (Figure 4a). The fluorescence lifetime had a slight decline with the protonation process, 3.58 ns for 44BD‐H crystal and 3.92 ns for 44BD crystal (Supporting Information S1: Figure S4). Meanwhile, when the 44BD molecule is protonated by hydrochloric acid, the Φ_FL_ decreases from 9.65% (44BD) to 0.91% (44BD‐H). Furthermore, the reason for the significant decrease in crystal Φ_FL_ can be attributed to the decrease in radiative transition rate (*K*_FL_) after crystal protonation. Moreover, the HOMO/LUMO orbitals of the 44BD and 44BD‐H molecules are simulated in Figure 4b, and both the HOMO and LUMO energy levels show an upshift after interaction with H^+^. The band gap between HOMO and LUMO of 44BD is 7.96 eV, and 4.22 eV for 44BD‐H. The smaller band gap of 44BD‐H coincided well with the red‐shift of the fluorescence emission peaks. Furthermore, the XRD results demonstrated that the intensity of the X‐ray diffraction peak of 44BD‐H decreases and the peak position also changes, indicating that their stacking methods are different (Supporting Information S1: Figure S8).

As shown in Figure 4c, the molecules exhibited two conformations in the 44BD crystal structure, with corresponding dihedral angles between the two pyridine rings of 18.73° and 34.69°. In the 44BD‐H crystal structure, the protonated molecule exhibited a unique conformation with a dihedral angle of 23.74° between the two pyridine rings (Figure 4d). Additionally, the lattice parameters summarized in Supporting Information S1: Table S3 indicate that the 44BD‐H single crystal has a looser packing structure than the 44BD single crystal. To better understand the various molecular packing motifs before and after protonation, intermolecular interactions in the 44BD and 44BD‐H single crystals were carefully analyzed. The two adjacent 44BD molecules exhibited a vertical stacking mode, leading to C‐H···N double hydrogen bond interactions. In the crystal structure, each 44BD molecule was surrounded by another four molecules, generating eight C‐H···N hydrogen bonds around it (Figure 4c). However, the molecules stack in parallel in the head‐to‐head mode along the b‐axis in the 44BD‐H single crystal. As shown in Figure 4d, two parallel pyridine cores partly overlapped, and the vertical distance between them was 3.575 Å, indicating intermolecular *π*–*π* stacking interactions. The smallest distance from N to H‐C was above 4.9 Å, meaning that no potential C‐H···N bonds form in the 44BD‐H single crystal.

The protonation of the pyridine groups not only changes the molecular conformation but also passivates the hydrogen bonds in the 44BD crystal structure. The reversible HCl/TEA stimulus‐response of the 44BD‐DITF and 44BD‐TFBA co‐crystals is also related to the interaction between the nitrogen atom in the pyridine group and the proton in the acid (Figure 4e,f). Fuming with HCl, protonated host (44BD) molecules dissociate from the gust (DITF or TFBA) molecules, breaking the halogen/hydrogen bonds in the co‐crystal structures. The sharp decrease in halogen/hydrogen bond interactions blocks the ISC process, leading to quenching of the RTP. Triethylamine can capture the protons in the co‐crystals and release neutral pyridine groups. The hydrogen/halogen bonds were reformed in the co‐crystal structures, resulting in the recovery of the RTP phenomenon.

## 3 CONCLUSION

In summary, we present two successful organic RTP co‐crystals that show reversible acid/base stimulus‐responsive RTP by incorporating the luminescent molecule 44BD and halogenated benzenes DITF and TFBA. Notably, the 44BD‐TFBA co‐crystal demonstrates a strong yellow afterglow with a remarkably high Φ_ISC_ of 99.64%, and a phosphorescence lifetime of 88.67 ms at 619 nm. Detailed analysis of the molecular packing structures and non‐covalent intermolecular interactions within the co‐crystals revealed that the abundant hydrogen and halogen bonds effectively suppressed non‐radiative transitions, thereby enhancing the RTP process. Furthermore, by alternately applying fuming HCl and TEA, we induced a protonation‐deprotonation transition in the pyridine groups of 44BD. Protonation disrupts the hydrogen and halogen bonds within the co‐crystals, resulting in phosphorescence quenching, while deprotonation reconstitutes these bonds, restoring the long RTP effect. Our study provides profound insights into the underlying mechanisms, thereby facilitating the tailored design of stimulus‐responsive RTP cocrystal materials. Additionally, the tunable changes in phosphorescence lifetime, emission color, and intensity under external stimuli could pave the way for the potential applications of RTP co‐crystal materials in time‐resolved bioimaging, time‐resolved security, and related fields.

## CONFLICT OF INTEREST STATEMENT

The authors declare no conflicts of interest.

## ETHICS STATEMENT

No animal or human experiments were involved in this study.

## Supporting information

Supporting Information S1

## Data Availability

The data that support the findings of this study are available from the corresponding author upon reasonable request.
