# Supplementary material for: Organic room temperature phosphorescence co‐crystal with reversible acid/base stimulus response
Source: Smart Mol. 2025 Jan 4;3(4):e20240054. doi: 10.1002/smo.20240054 (PMC12755218; doi:10.1002/smo.20240054)
Supplement: Supplementary file 1 — Supporting Information S1 [file SMO2-3-e20240054-s001.docx]

Supporting information

**Organic Room Temperature Phosphorescence Co-Crystal with Reversible Acid/base Stimulus Response**

Chenchen Zhang, Xingjia Jiang, Can Wang*, Zhaoyang Liu, Bin Xu*, Wenjing Tian

State Key Laboratory of Supramolecular Structure and Materials, Jilin University, Changchun 130000, Jilin Province, China

**Corresponding authors,** E-mail: [xubin@jlu.edu.cn](mailto:xubin@jlu.edu.cn); [canwang@jlu.edu.cn](mailto:canwang@jlu.edu.cn)

1. **General information**

The 4,4'-bipyridine (44BD), 1,4-diiodotetrafluorobenzene (DITF), and 4-bromo-2,3,5,6-tetrafluorobenzoic acid (TFBA) are purchased from TCI. All solvents are of commercial grade and are used without further purification.

**2. Crystal Growth:**

**44BD-DITF:** The mixed powder of 44BD and DITF is weighed with a stoichiometric ratio of 2:1 and dissolved in a mixed solvent of acetone and methanol with a volume ratio of 2:1. At room temperature and in dark conditions, the solution is allowed to stand still and the solvent slowly evaporated, ultimately resulting in blue rod-shaped crystals.

**44BD-TFBA:** The mixed powder of 44BD and TFBA is weighed with a stoichiometric ratio of 2:1 and dissolved in a mixed solvent of dichloromethane and methanol with a volume ratio of 2:1. At room temperature and in dark conditions, the solution is allowed to stand still and the solvent slowly evaporated, resulting in yellow-green block-shaped crystals.

**44BD:** The 44BD single crystal is prepared by the solvent vapor method from a mixed solution of dichloromethane and methanol.

**44BD-H:** The 44BD-H crystal is obtained by mixing 1-2 drops of dilute hydrochloric acid into a dichloromethane/methanol solution containing 44BD, then placing a small sample bottle containing the solution into a larger bottle, adding methanol to the larger bottle, allowing it to stand, and slowly evaporating the solvent.

**3. Crystal structure analysis:**

Crystal diffraction data are obtained with graphite-monochromated Mo Kα radiation on a Rigaku RAXISRAPID diffractometer. The structure is solved by direct methods using the SHELX program and refined with least-squares methods. The crystallographic data for the structures are given in Tables S2, and S3.

**4. Optical Properties:**

A steady-state transient fluorescence spectrometer (model: FLS980, manufacturer: Edinburgh Instruments, light source: 100 Hz xenon lamp) is used for the fluorescence spectrum. The steady-state transient fluorescence spectrometer (model: FLS920, manufacturer: Edinburgh Instruments, UK) is used for the variable temperature spectrum and low-temperature spectrum. The test is conducted in a vacuum with liquid nitrogen added at low temperatures, and heating is controlled by the program and stabilized at the target temperature for 2 minutes. The time-resolved fluorescence spectra are also collected on an FLS980 spectrometer when a laser is used (model: EPL-375, manufacturer: Edinburgh Instruments, wavelength: 378.8 nm, pulse width: 68.9 ps). A microsecond light source is used for testing the long life. For the test of fluorescence quantum efficiency, a steady-state transient fluorescence spectrometer (model: FLS980, manufacturer: Edinburgh Instruments) is chosen, and the measured efficiency is absolute efficiency. The solid is tested using the integrating sphere.

**5. X-ray diffraction (XRD):**

The collection of powder X-ray diffraction data is carried out using an X-ray diffractometer (model: Smart Lab, manufacturer: Rigaku Company, Japan, copper target, wavelength 1.5418 Å).

**6. Theoretical calculations:**

All density functional theory (DFT) calculations involved in this paper are completed using the Gaussian16 package. The molecular configurations used for theoretical calculations in this paper are directly obtained from the crystal structure. The functional and basis set used for the calculation of the frontier orbital distribution are m062x/6-31g (d, p). The functional and basis set used for the calculation of the vertical excitation energy of the ground and excited states are b3lyp/6-31g (d, p). The calculation of the SOC constant is carried out using the BDF program package, and the functional and basis set are b3lyp/6-31g (d, p). The Hirschfeld surface analysis is calculated using Crystal Explorer software.


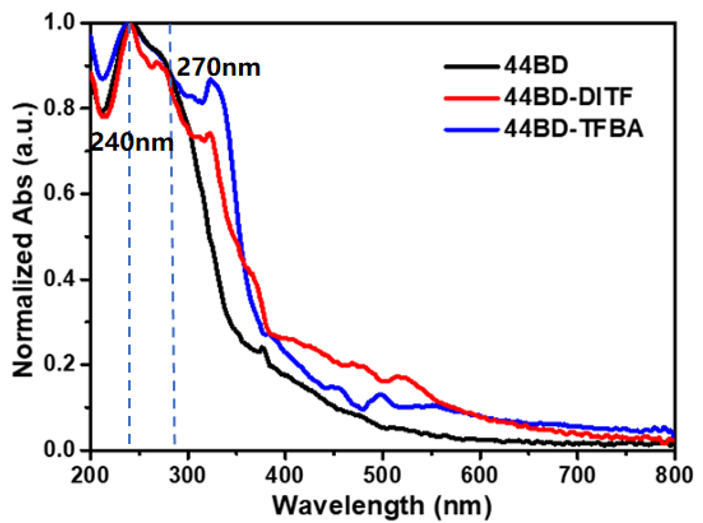


**Figure S1.** Solid UV-visible absorption spectra of 44BD, 44BD-DITF, and 44BD-TFBA crystals.

All crystals have absorption peaks at 240 nm and 270 nm. Compared to the 44BD single crystal, the absorption spectra of 44BD-DITF and 44BD-TFBA crystals show an obvious absorption after 400 nm due to the guest molecules’ participation.


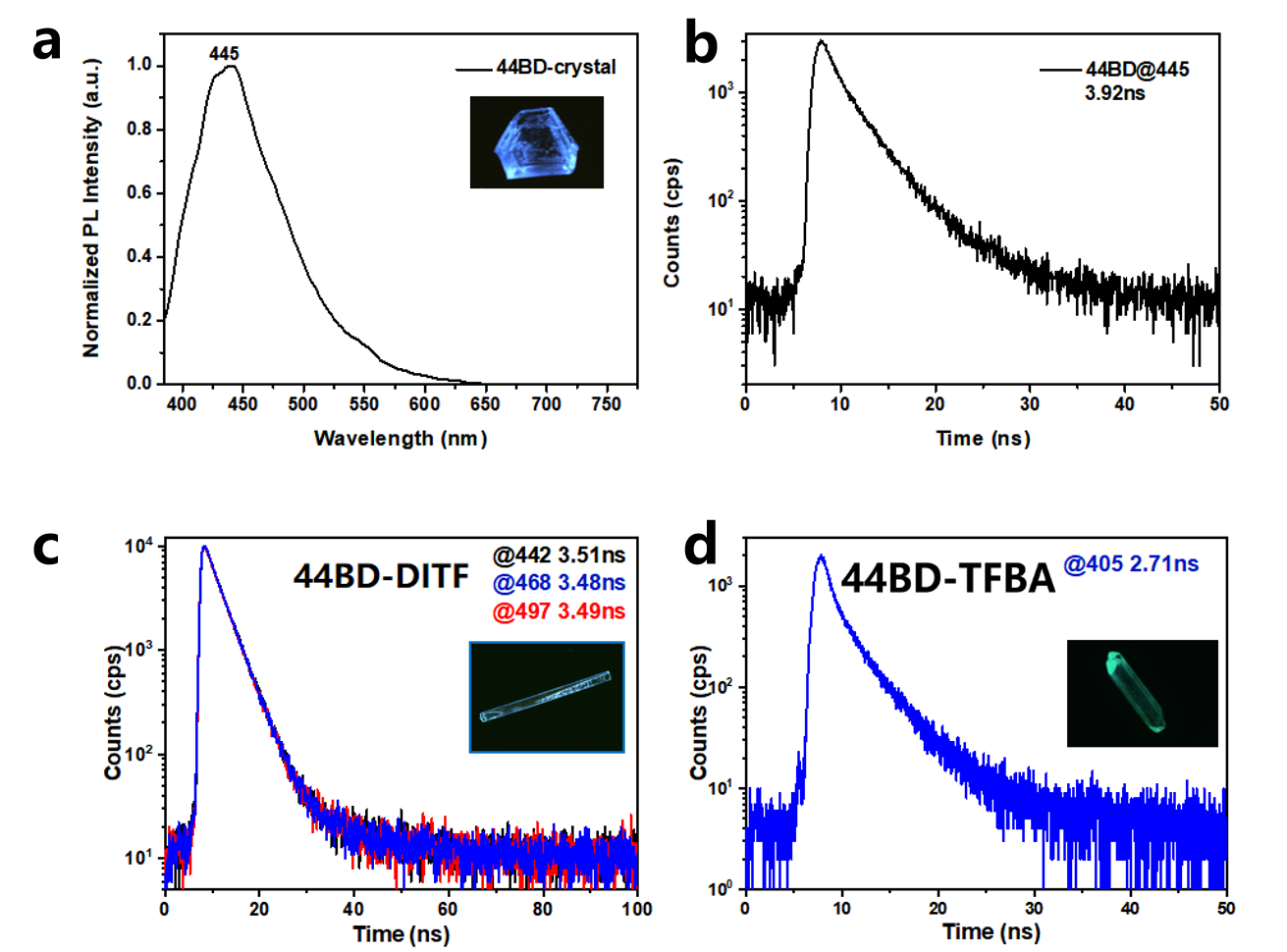


**Figure S2.** (a) Photoluminescence spectra of 44BD crystal. (b) Fluorescence lifetime of 44BD crystal at 445 nm. (c) Fluorescence lifetime of 44BD-DITF co-crystal at 442 nm, 468 nm, and 497 nm. (d) Fluorescence lifetime of 44BD-TFBA co-crystal at 405 nm.

The 44BD crystal exhibits fluorescence emission at a wavelength of 445 nm, with a lifetime of 3.92 ns and a fluorescence efficiency of 9.65%. For 44BD-DITF co-crystals, the lifetime of the main fluorescence peak at 468 nm is 3.48 ns, and there are two shoulder peaks with lifetimes of 3.51 ns at 442 nm and 3.49 ns at 497 nm. For 44BD-TFBA co-crystals, the fluorescence lifetime at 405 nm is 2.71 ns.


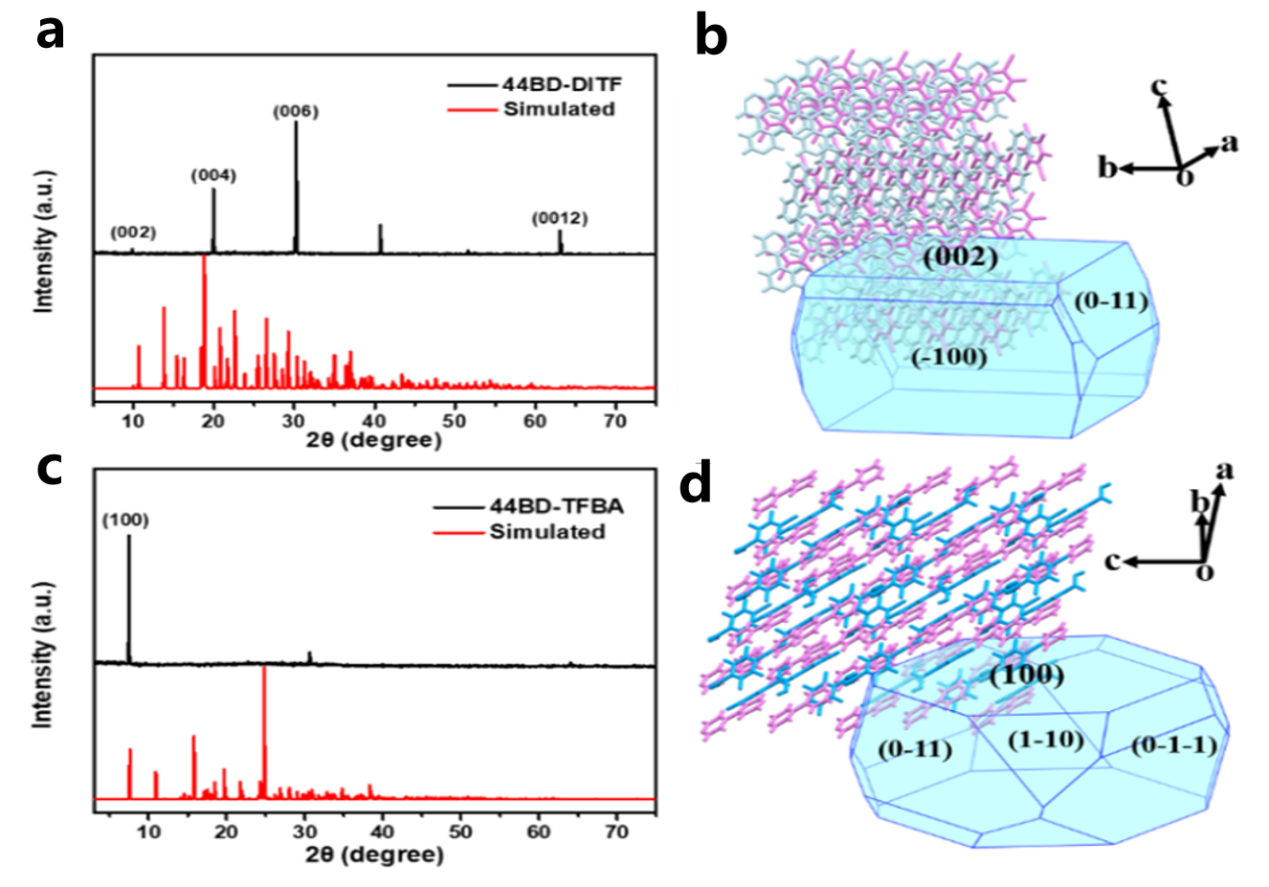


\

**Figure S3.** (a) The XRD of the 44BD-DITF co-crystal; (b) Crystal growth morphology predicted by theoretical calculations of 44BD-DITF co-crystals. (c) The XRD of the 44BD-TFBA co-crystal; (d) Crystal growth morphology predicted by theoretical calculations of 44BD-TFBA co-crystals.

The three diffraction peaks of the 44BD-DITF co-crystal belong to the (002) crystal plane, (004) crystal plane, and (006) crystal plane respectively. Through the prediction of the thermodynamic stability of co-crystals simulated by Mercury software, the crystal surface belongs to the (002) crystal plane, indicating the crystal grows along the b-axis direction. The 44BD-TFBA crystal exhibits two diffraction peaks. By comparing with the simulated XRD, it is found that the first diffraction peak belongs to the (100) crystal plane. At the same time, the 44BD-TFBA crystal grows along the c-axis direction and the crystal surface belongs to the (100) crystal plane.


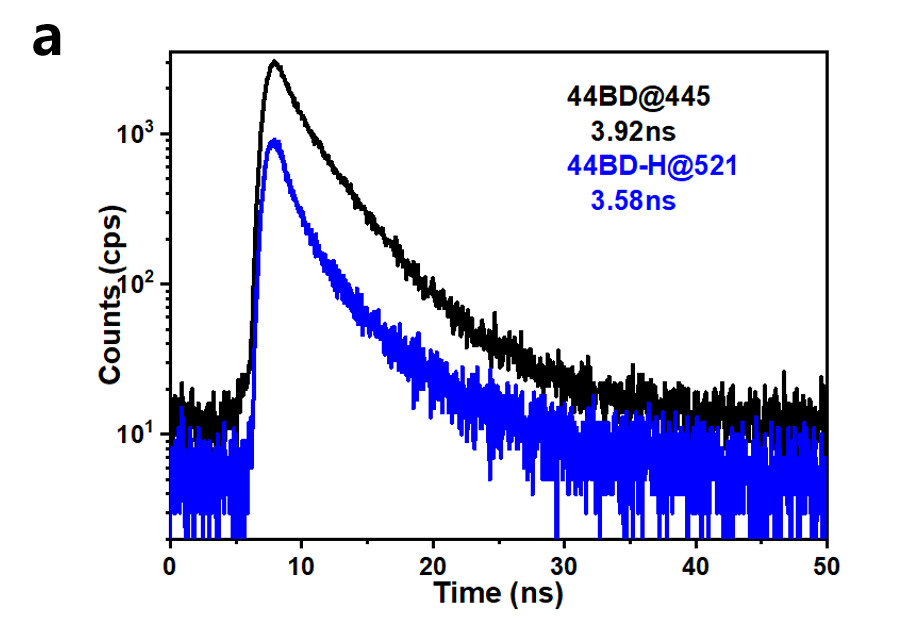


**Figure S4.** The fluorescence lifetime of the 44BD crystal at 465 nm and the 44BD-H crystal at 521 nm.

The fluorescence lifetime is 3.92 ns at 445 nm for 44BD crystals and 3.58 ns at 521 nm for 44BD-H crystals.


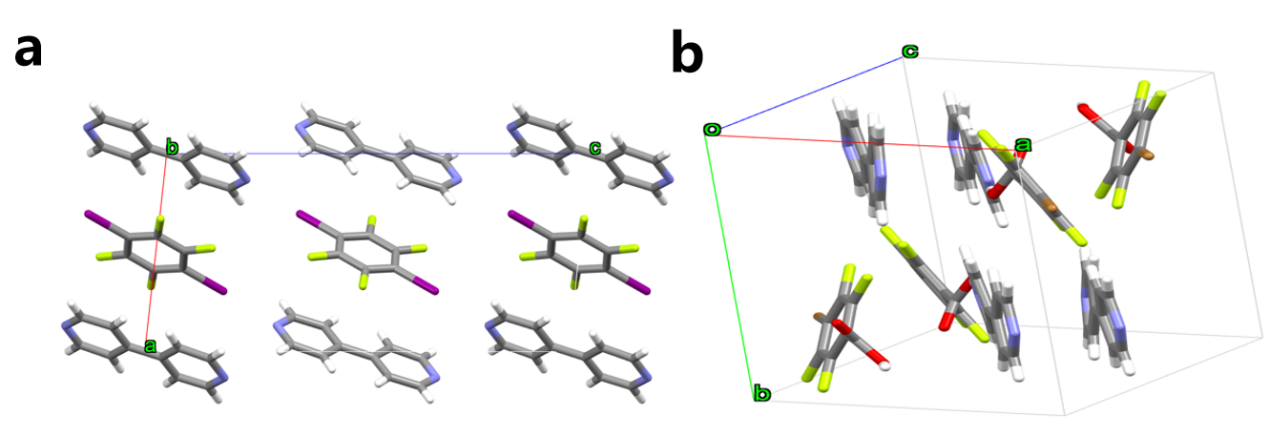


**Figure S5.** (a) Single unit cell structure of the 44BD-DITF crystal. (b) Single unit cell structure of the 44BD-TFBA crystal. Specific parameters are shown in Table S2.


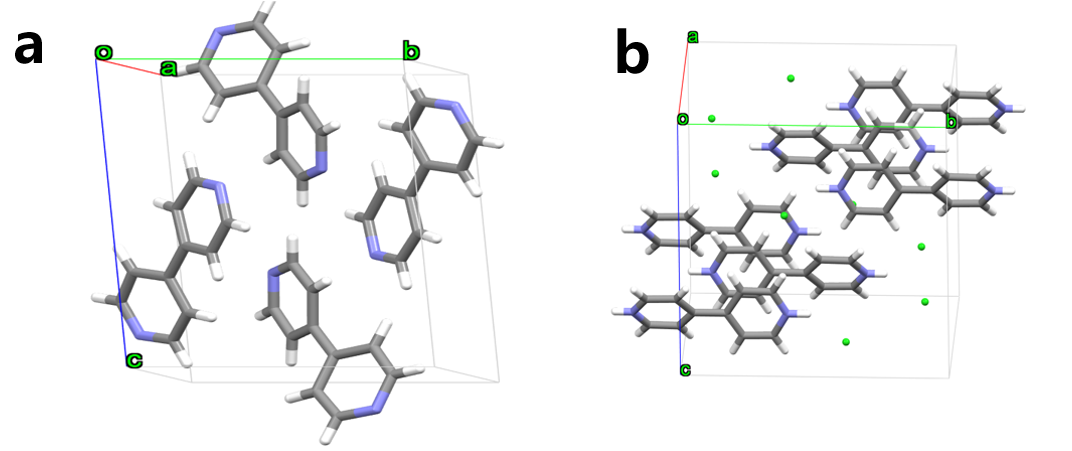


**Figure S6.** (a) Single unit cell structure of the 44BD crystal. (b) Single unit cell structure of the 44BD-H crystal. Specific parameters are shown in Table S3.


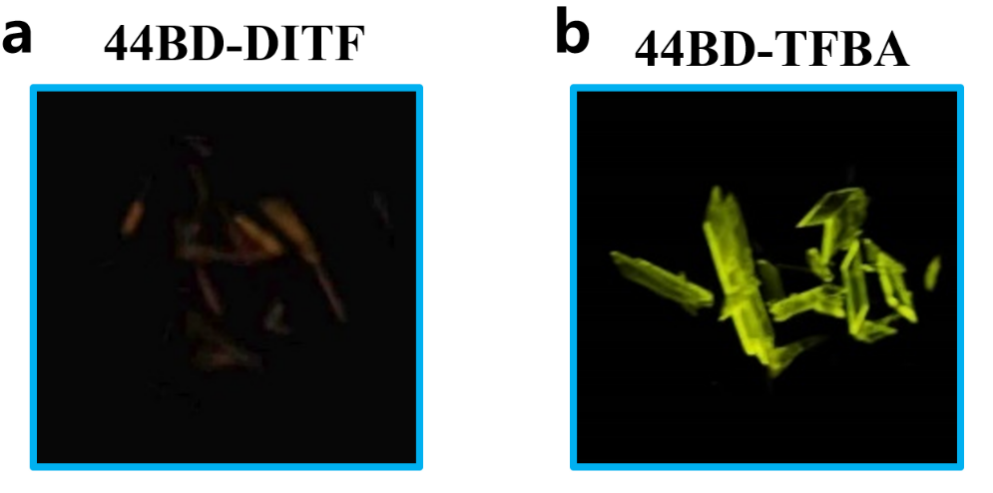


**Figure S7.** The phosphorescence images of (a) 44BD-DITF (b) 44BD-TFBA co-crystals before and after HCl/TEA treatment.

Figure S7a and b show the phosphorescence images before and after acid fumigation of (a) 44BD-DITF (b) 44BD-TFBA respectively. After acid fumigation, the phosphorescence almost disappears, so no corresponding phosphorescence image is available.

**
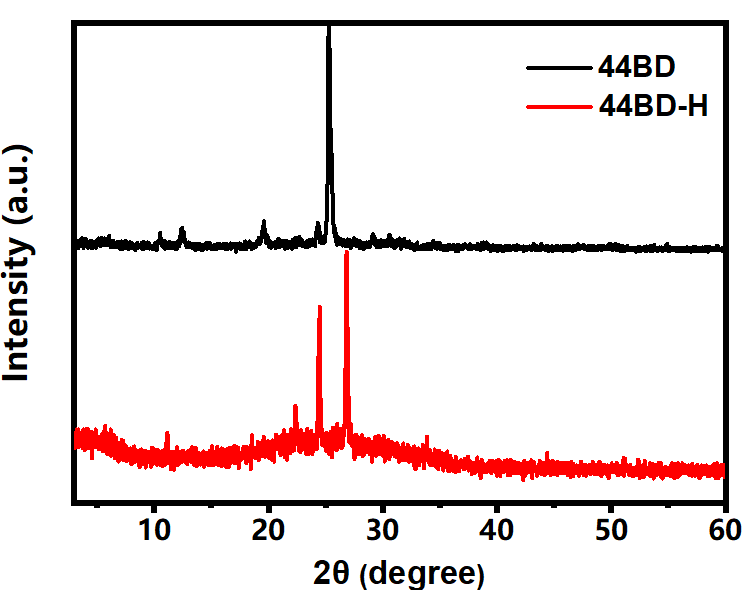
**

**Figure S8.** The XRD images of 44BD and 44BD-H crystals.

The results demonstrated the intensity of the X-ray diffraction peak of 44BD-H decreases and the peak position also changes, indicating that their stacking methods are different.

**Table S1:** Photophysical parameters of the 44BD, 44BD-DITF, and 44BD-TFBA crystals.

| Sample | λ_FL_  (nm) | λ_Ph_  (nm) | Φ_Fl_  (%) | Φ_Ph_  (%) | Φ_ISC_  (%) | τ_Fl_  (ns) | τ_Ph_  (ms) | k_Fl_  (s^-1^) | k_ISC_  (s^-1^) | k_Ph_  (s^-1^) | k_Ph,nr_  (s^-1^) |
| --- | --- | --- | --- | --- | --- | --- | --- | --- | --- | --- | --- |
| 44BD | 445 | - | 9.65 | - | 90.35 | 3.92 | - | 2.46× 10^7^ | 2.30× 10^8^ | - | - |
| 44BD-DITF | 468 | 597 | 11.93 | 1.46 | 88.07 | 3.48 | 18.08 | 3.43× 10^7^ | 2.53× 10^8^ | 0.92 | 54.39 |
| 44BD-TFBA | 405 | 529 | 0.36 | 1.56 | 99.64 | 2.71 | 77.15 | 1.33× 10^8^ | 3.68× 10^10^ | 0.20 | 12.76 |


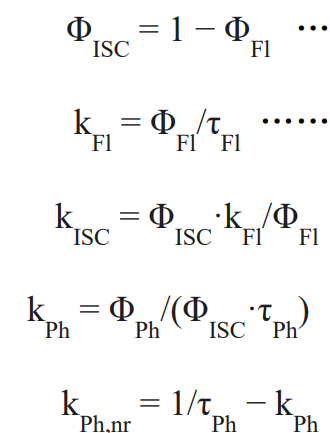


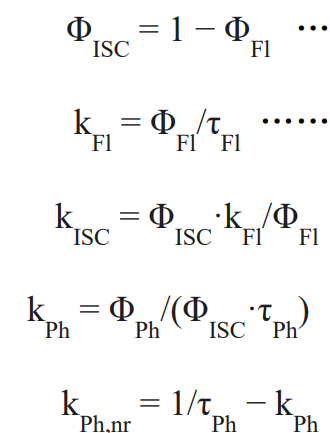


K_ph,nr_ refers to the non-radiative transition process from T_1_ to S_0_; kPh refers to the radiative transition process from T_1_ to S_0_; KISC refers to the intersystem crossing (ISC) from S_1_ to T_6_; kFl refers to the radiative transition process from S_1_ to S_0_;


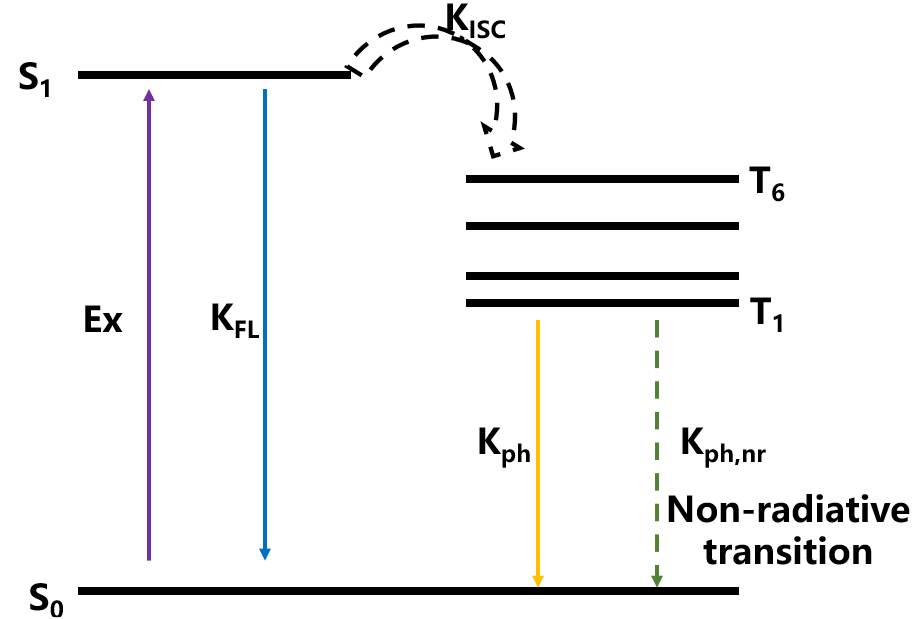


**Table S2:** 44BD-DITF and 44BD-TFBA crystal structures and refinement parameters.

| Name | 44BD-DITF | 44BD-TFBA |
| --- | --- | --- |
| empirical formula | C_32_H_16_F_8_I_4_N_4_ | C_17_H_9_BrF_4_N_2_O_2_ |
| formula wt | 1116.09 | 429.17 |
| T, K | 98 (2) | 100 |
| crystal system, space group | P21/c | P21/c |
| a, Å | 8.3565(5) | 11.9844(6) |
| b, Å | 5.7205(3) | 11.2178(6) |
| c, Å | 17.7609(10) | 12.4960(6) |
| α, deg | 90 | 90 |
| β, deg | 96.329(2) | 103.480(2) |
| γ, deg | 90 | 90 |
| V, Å^3^ | 843.86(8) | 1633.66(14) |
| Z | 1 | 4 |
| F (000) | 520.0 | 848.0 |
| density, Mg/m^3^ | 2.196 | 1.745 |
| Absorption coefficient, mm^-1^ | 3.767 | 2.574 |
| θ range, deg | 3.549-27.480 | 2.520-27.482 |
| no. of reflections collected | 16706 | 32218 |
| no. of unique reflections | 1936 | 3745 |
| R (int) | 0.0237 | 0.0286 |
| Good-of-fit on F^2^ | 1.079 | 1.026 |
| R1 [I>2σ(I)] | 0.0223 | 0.0260 |
| wR2 [I>2σ(I)] | 0.0515 | 0.0620 |
| R1 (all data) | 0.0256 | 0.0394 |
| wR_2_ (all data) | 0.0536 | 0.0682 |

**Table S3:** 44BD and 44BD-H crystal structures and refinement parameters.

| Name | 44BD | 44BD-H |
| --- | --- | --- |
| empirical formula | C_20_ H_16_ N_4_ | C_10_ H_10_ Cl_2_ N_2_ |
| formula wt | 312.37 | 229.10 |
| T, K | 291(2) | 98(2) |
| crystal system, space group | P-1 | I2/c |
| a, Å | 8.900(7) | 7.155(2) |
| b, Å | 8.853(7) | 12.109(3) |
| c, Å | 11.033(7) | 11.987(4) |
| α, deg | 85.335(19) | 90 |
| β, deg | 85.42(2) | 101.03(2) |
| γ, deg | 77.91(3) | 90 |
| V, Å^3^ | 845.4(11) | 1019.4(5) |
| Z | 2 | 4 |
| F (000) | 328.0 | 472.0 |
| density, Mg/m^3^ | 1.227 | 1.493 |
| Absorption coefficient, mm^-1^ | 0.075 | 0.595 |
| θ range, deg | 2.897-27.483 | 3.08-27.50 |
| no. of reflections collected | 30165 | 11510 |
| no. of unique reflections | 3911 | 1178 |
| R (int) | 0.0369 | 0.0329 |
| Good-of-fit on F^2^ | 1.029 | 1.076 |
| R1 [I>2σ(I)] | 0.0496 | 0.0315 |
| wR2 [I>2σ(I)] | 0.1233 | 0.0755 |
| R1 (all data) | 0.0725 | 0.0394 |
| wR_2_ (all data) | 0.1425 | 0.0794 |

**Table S4:** The intermolecular interactions in the 44BD-DITF co-crystal.

| Type | Distance(Å) | Angle(°) |
| --- | --- | --- |
| C-N…I | 2.861 | 177.08 |
| C-H…F | 2.598 | 164.77 |
| C-H…F | 2.647 | 167.60 |
| C-H…N | 1.908 | 178.05 |

Through Hirshfeld surface analysis, the statistical results of halogen bonds and hydrogen bonds in 44BD-DITF co-crystals are shown in Table S4.

**Table S5:** The intermolecular interactions in the 44BD-TFBA co-crystal.

| Type | Distance(Å) | Angle(°) |
| --- | --- | --- |
| C-N…Br | 2.924 | 175.44 |
| C-N…O | 1.708 | 179.09 |
| C-H…O | 2.470 | 163.22 |
| C-H…O | 2.526 | 144.70 |
| C-H…F | 2.795 | 122.54 |
| C-H…F | 2.843 | 167.89 |
| C-H…F | 2.750 | 148.72 |
| C-H…F | 2.501 | 125.61 |
| C-H…F | 2.764 | 162.17 |

Through Hirshfeld surface analysis, the statistical results of halogen bonds and hydrogen bonds in 44BD-TFBA co-crystals are shown in Table S5.

**Table S6:** Singlet and triplet energy states of the 44BD molecule in 44BD-DITF and 44BD-TFBA.

| Name | 44BD-DITF | 44BD-TFBA |
| --- | --- | --- |
| S_1_ | 4.0739 | 4.1312 |
| T_1_ | 3.3624 | 3.3462 |
| T_2_ | 3.6260 | 3.5640 |
| T_3_ | 3.7474 | 3.6845 |
| T_4_ | 3.7519 | 3.7498 |
| T_5_ | 4.0187 | 4.0067 |
| T_6_ | 4.0546 | 4.1293 |

The main transition orbitals from singlet to triplet states in 44BD-DITF and 44BD-TFBA co-crystals are S1→T1, S1→T2, S1→T3, S1→T4, S1→T5, and S1→T6.
